# Methyl 2-[(2-chloro­quinolin-3-yl)(hy­droxy)meth­yl]acrylate

**DOI:** 10.1107/S1600536813014050

**Published:** 2013-05-31

**Authors:** T. Anuradha, J. Srinivasan, P. R. Seshadri, M. Bakthadoss

**Affiliations:** a Post Graduate and Research Department of Physics, Agurchand Manmull Jain College, Chennai 600 114, India; b Department of Organic Chemistry, University of Madras, Guindy Campus, Chennai 600 025, India

## Abstract

There are two independent mol­ecules (*A* and *B*) in the asymmetric unit of the title compound, C_14_H_12_ClNO_3_. The mean planes of the methyl ester unit (C_meth­yl_—O—C=O; r.m.s. deviation = 0.051 Å for mol­ecule *A* and 0.016 Å for mol­ecule *B*) and the chloro­quilonine ring system (r.m.s. deviation = 0.023 Å for mol­ecule *A* and 0.014 Å for mol­ecule *B*) form dihedral angles of 63.5 (1)° in mol­ecule *A* and 78.1 (1)° in mol­ecule *B*. The main difference between the two independent mol­ecules is reflected in the (H)O—C—C=C(H_2_) torsion angle which is −109.7 (2)° in mol­ecule *A* and 10.6 (2)° in mol­ecule *B*. An intra­molecular O—H⋯O hydrogen bond is observed in mol­ecule *A*. In the crystal, mol­ecules *A* and *B* are linked into pairs *via* bifurcated O—H⋯(N,Cl) hydrogen bonds and a weak C—H⋯O hydrogen bond links pairs of mol­ecules into chains along [100].

## Related literature

For the biological activity of quilonine compounds, see: Biavatti *et al.* (2002 ▶); Towers *et al.* (1981 ▶); Shen *et al.* (1999 ▶). For their luminescent properties, see: Montes *et al.* (2006 ▶). For applications of acrylate compounds, see: Bhatia *et al.* (2007 ▶); Sharma (2011 ▶). For conformational aspects of methyl esters, see: Dunitz & Schweizer (1982 ▶). For resonance effects in acrylates, see: Merlino (1971 ▶); Varghese *et al.* (1986 ▶).
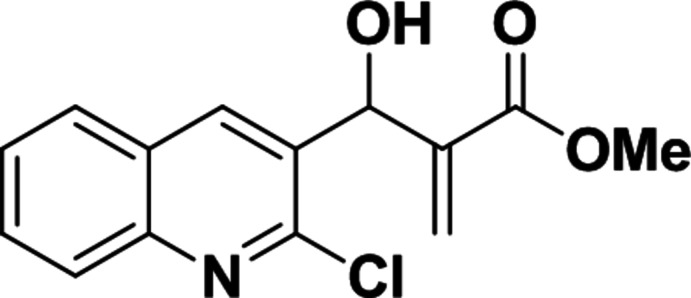


## Experimental

### 

#### Crystal data

C_14_H_12_ClNO_3_
*M* *_r_* = 277.70
Triclinic, 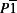

*a* = 9.2614 (4) Å
*b* = 11.0309 (4) Å
*c* = 13.8161 (6) Å
α = 102.557 (2)°
β = 100.646 (2)°
γ = 103.704 (2)°
*V* = 1296.29 (9) Å^3^
*Z* = 4
Mo *K*α radiation
μ = 0.30 mm^−1^
*T* = 293 K
0.35 × 0.30 × 0.25 mm

#### Data collection

Bruker SMART APEXII diffractometer
14402 measured reflections
4466 independent reflections
3767 reflections with *I* > 2σ(*I*)
*R*_int_ = 0.022

#### Refinement

*R*[*F* ^2^ > 2σ(*F* ^2^)] = 0.032
*wR*(*F* ^2^) = 0.093
*S* = 1.03
4466 reflections
344 parameters
H-atom parameters constrained
Δρ_max_ = 0.29 e Å^−3^
Δρ_min_ = −0.25 e Å^−3^

### 

Data collection: *APEX2* (Bruker, 2008 ▶); cell refinement: *SAINT* (Bruker, 2008 ▶); data reduction: *SAINT*; program(s) used to solve structure: *SHELXS97* (Sheldrick, 2008 ▶); program(s) used to refine structure: *SHELXL97* (Sheldrick, 2008 ▶); molecular graphics: *ORTEP-3 for Windows* (Farrugia, 2012 ▶) and *PLATON* (Spek, 2009 ▶); software used to prepare material for publication: *SHELXL97*, *PLATON* and *publCIF* (Westrip, 2010 ▶).

## Supplementary Material

Crystal structure: contains datablock(s) I, global. DOI: 10.1107/S1600536813014050/lh5607sup1.cif

Structure factors: contains datablock(s) I. DOI: 10.1107/S1600536813014050/lh5607Isup2.hkl

Supplementary material file. DOI: 10.1107/S1600536813014050/lh5607Isup3.cml

Additional supplementary materials:  crystallographic information; 3D view; checkCIF report

## Figures and Tables

**Table 1:** Hydrogen-bond geometry (Å, °)

*D*—H⋯*A*	*D*—H	H⋯*A*	*D*⋯*A*	*D*—H⋯*A*
O1*A*—H1*A*⋯O2*A*	0.82	2.24	2.8372 (19)	130
O1*B*—H1*B*⋯Cl1*A* ^i^	0.82	2.79	3.5040 (12)	147
O1*B*—H1*B*⋯N1*A* ^i^	0.82	2.16	2.8609 (17)	144
C5*A*—H5*A*⋯O1*B* ^ii^	0.93	2.56	3.451 (2)	162

Symmetry codes: (i) 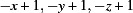; (ii) 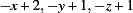.

**Fig. 1.**
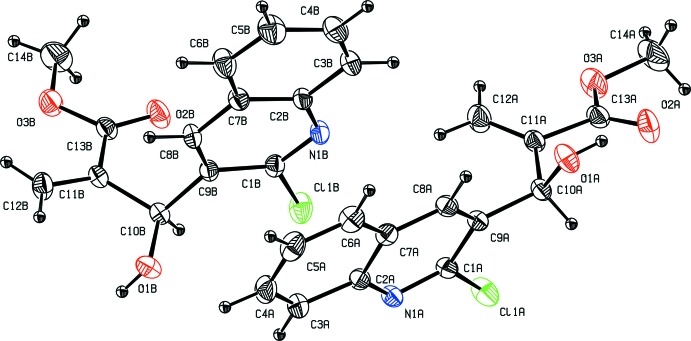
The asymmetric unit of the title compound showing 30% probability displacement ellipsoids. H atoms are shown as spheres of arbitrary radius.

**Fig. 2.**
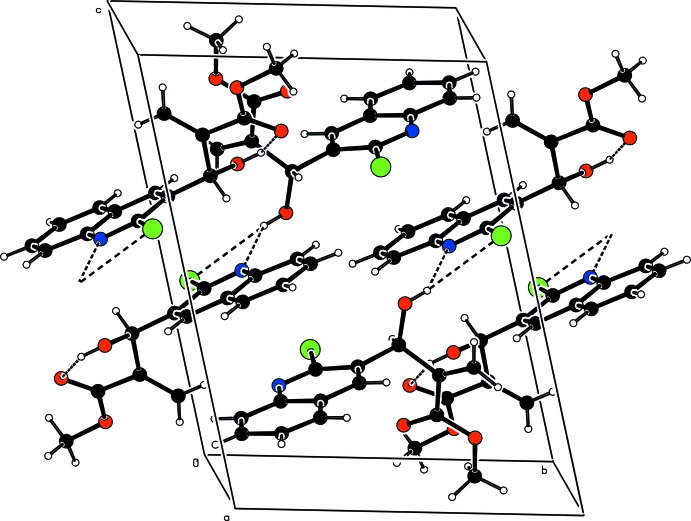
Part of the crystal structure with hydrogen bonds shown as dashed lines.

## supplementary crystallographic information

### Comment

The quinoline ring is found in compounds with antifungal (Biavatti *et al.*, 2002), antibacterial (Towers *et al.*, 1981) and anticancer (Shen *et al.*, 1999) properties. Quinoline derivatives have been used for their luminescent properties as organic light-emitting diode (OLED) materials (Montes *et al.*, 2006). Methyl acrylate is an ingredient used in many fragrances and decorative cosmetics (Bhatia *et al.*, 2007; Sharma, 2011). In view of the potential importance of the title compound its crystal structure is presented herein.

The asymmetric unit of the title compound contains the two independent molecules, A and B (Fig. 1). The dihedral angle between the mean plane of methyl ester unit (C13/C14/O2/O3, r.m.s deviation = -0.051 Å for A and -0.016 Å for B) and the chloroquilonin ring system (C1—C9/N1/Cl1, r.m.s deviation = 0.023 Å for A and -0.014 Å for B) is 63.5 (1)° in molecule A and 78.6 (1)° in molecule B. The main difference between the two independent molecules is reflected in the O1—C10—C11—C12 torsion angle which is -109.7 (2)° in molecule A and 10.6 (2)° in molecule B.

The methyl ester moiety adopts an extended conformation as reflected by the torsion angles for C11—C13—C14—O3 = 177.7 (2)° in A and 178.4 (1)° in B. The extended conformation is supported by the fact that the bond angles involving the carbonyl O atoms are invariably expanded (Dunitz & Schweizer, 1982). The significant difference in the bond lengths of the C13—O3 = 1.322 (3) Å (A) 1.331 (3) Å (B) versus C14—O3 = 1.447 (3) Å (A) and 1.447 (2) Å (B) can be attributed to a partial contribution from the O^-^–C═O^+^–C resonance structure of the O2—C13—O3—C14 group (Merlino, 1971). This feature, commonly observed in the carboxylic ester group of these substituents in various compounds has been shown to give average values of 1.340 Å and 1.447 Å respectively for these bonds (Varghese *et al.*, 1986).

In the crystal molecule A and B are linked into pairs via bifurcated O—H···(N,Cl) hydrogen bonds (Fig. 2) and a weak C—H···O hydrogen bond links pairs of molecules into chains along [100].

### Experimental

A mixture of 2-chloroquinoline-3-carbaldehyde (0.1 g, 0.52 mmol), methyl acrylate (0.071 ml, 0.78 mmol), and DABCO (0.017 g, 0.15 mmol), was kept at room temperature for 7 days. After completion of the reaction (indicated by TLC), the reaction mixture was extracted with ethylacetate (3 τimes 15 ml). The combined organic layer subsequently washed with dil.HCl and dried over anhydrous Na_2_SO_4_. The solvent was evaporated under reduced pressure. The crude product was obtained and purified by column chromatography eluting with 8% ethylacetate in hexane afforded the alcohol methyl 2-((2-chloroquinolin-3-yl)(hydroxy)methyl)acrylate as a colourless solid. X-ray quality crystals were obtained by slow evaporation of a solution of the title compound in ethylacetate.

### Refinement

Hydrogen atoms were positioned geometrically and allowed to ride on their parent atoms, with C—H = 0.93-0.98 Å, O—H = 0.82° and U_iso_(H) = 1.5U_eq_(C) for methyl and hydroxyl H atoms and 1.2U_eq_(C) for other H atoms.

### Crystal data

**Table:**

C_14_H_12_ClNO_3_	*Z* = 4
*M**_r_* = 277.70	*F*(000) = 576
Triclinic, *P*1	*D*_x_ = 1.423 Mg m^−^^3^
Hall symbol: -P 1	Mo *K*α radiation, λ = 0.71073 Å
*a* = 9.2614 (4) Å	Cell parameters from 4551 reflections
*b* = 11.0309 (4) Å	θ = 2.0–25.0°
*c* = 13.8161 (6) Å	µ = 0.30 mm^−^^1^
α = 102.557 (2)°	*T* = 293 K
β = 100.646 (2)°	Block, colourless
γ = 103.704 (2)°	0.35 × 0.30 × 0.25 mm
*V* = 1296.29 (9) Å^3^	

### Data collection

**Table:**

Bruker SMART APEXII diffractometer	3767 reflections with *I* > 2σ(*I*)
Radiation source: fine-focus sealed tube	*R*_int_ = 0.022
Graphite monochromator	θ_max_ = 25.0°, θ_min_ = 2.0°
ω and φ scans	*h* = −11→10
14402 measured reflections	*k* = −12→13
4466 independent reflections	*l* = −16→16

### Refinement

**Table:**

Refinement on *F*^2^	Secondary atom site location: difference Fourier map
Least-squares matrix: full	Hydrogen site location: inferred from neighbouring sites
*R*[*F*^2^ > 2σ(*F*^2^)] = 0.032	H-atom parameters constrained
*wR*(*F*^2^) = 0.093	*w* = 1/[σ^2^(*F*_o_^2^) + (0.0462*P*)^2^ + 0.4048*P*] where *P* = (*F*_o_^2^ + 2*F*_c_^2^)/3
*S* = 1.03	(Δ/σ)_max_ < 0.001
4466 reflections	Δρ_max_ = 0.29 e Å^−^^3^
344 parameters	Δρ_min_ = −0.25 e Å^−^^3^
0 restraints	Extinction correction: *SHELXL97* (Sheldrick, 2008), Fc^*^=kFc[1+0.001xFc^2^λ^3^/sin(2θ)]^-1/4^
Primary atom site location: structure-invariant direct methods	Extinction coefficient: 0.0040 (11)

### Special details

**Table:**

Geometry. All e.s.d.'s (except the e.s.d. in the dihedral angle between two l.s. planes) are estimated using the full covariance matrix. The cell e.s.d.'s are taken into account individually in the estimation of e.s.d.'s in distances, angles and torsion angles; correlations between e.s.d.'s in cell parameters are only used when they are defined by crystal symmetry. An approximate (isotropic) treatment of cell e.s.d.'s is used for estimating e.s.d.'s involving l.s. planes.
Refinement. Refinement of *F*^2^ against ALL reflections. The weighted *R*-factor *wR* and goodness of fit *S* are based on *F*^2^, conventional *R*-factors *R* are based on *F*, with *F* set to zero for negative *F*^2^. The threshold expression of *F*^2^ > σ(*F*^2^) is used only for calculating *R*-factors(gt) *etc*. and is not relevant to the choice of reflections for refinement. *R*-factors based on *F*^2^ are statistically about twice as large as those based on *F*, and *R*- factors based on ALL data will be even larger.

### Fractional atomic coordinates and isotropic or equivalent isotropic displacement parameters (Å^2^)

**Table:**

	*x*	*y*	*z*	*U*_iso_*/*U*_eq_	
Cl1A	0.14491 (5)	0.05724 (5)	0.40164 (4)	0.05417 (16)	
O1A	0.36327 (14)	−0.23933 (11)	0.28015 (10)	0.0504 (3)	
H1A	0.3051	−0.3109	0.2473	0.076*	
O2A	0.05490 (17)	−0.36860 (13)	0.16879 (12)	0.0655 (4)	
O3A	−0.03759 (16)	−0.26279 (14)	0.06395 (11)	0.0626 (4)	
N1A	0.42615 (15)	0.19802 (13)	0.45982 (10)	0.0356 (3)	
C1A	0.33952 (18)	0.08303 (15)	0.40781 (12)	0.0338 (4)	
C2A	0.58039 (18)	0.22445 (15)	0.46705 (12)	0.0342 (4)	
C3A	0.6764 (2)	0.35052 (17)	0.51704 (13)	0.0443 (4)	
H3A	0.6352	0.4153	0.5445	0.053*	
C4A	0.8297 (2)	0.37743 (19)	0.52515 (15)	0.0520 (5)	
H4A	0.8929	0.4611	0.5583	0.062*	
C5A	0.8944 (2)	0.2818 (2)	0.48466 (15)	0.0521 (5)	
H5A	0.9999	0.3017	0.4921	0.062*	
C6A	0.8037 (2)	0.16025 (19)	0.43468 (14)	0.0456 (4)	
H6A	0.8474	0.0973	0.4073	0.055*	
C7A	0.64369 (18)	0.12781 (16)	0.42366 (12)	0.0350 (4)	
C8A	0.54251 (18)	0.00498 (16)	0.36909 (12)	0.0356 (4)	
H8A	0.5817	−0.0604	0.3401	0.043*	
C9A	0.38875 (18)	−0.02014 (15)	0.35779 (12)	0.0321 (3)	
C10A	0.27692 (18)	−0.14936 (15)	0.29730 (12)	0.0352 (4)	
H10A	0.2067	−0.1775	0.3385	0.042*	
C11A	0.18355 (18)	−0.14703 (15)	0.19609 (12)	0.0368 (4)	
C12A	0.2087 (2)	−0.05086 (19)	0.15361 (15)	0.0547 (5)	
H12A	0.1496	−0.0596	0.0889	0.066*	
H12B	0.2855	0.0259	0.1883	0.066*	
C13A	0.0617 (2)	−0.27074 (17)	0.14275 (14)	0.0433 (4)	
C14A	−0.1553 (3)	−0.3815 (2)	0.00547 (18)	0.0770 (7)	
H14A	−0.2208	−0.3645	−0.0494	0.116*	
H14B	−0.2151	−0.4134	0.0493	0.116*	
H14C	−0.1082	−0.4451	−0.0223	0.116*	
Cl1B	0.34205 (5)	0.35070 (5)	0.27784 (4)	0.05394 (16)	
N1B	0.52758 (16)	0.22798 (12)	0.21855 (10)	0.0386 (3)	
O1B	0.71157 (13)	0.63179 (11)	0.42717 (8)	0.0422 (3)	
H1B	0.7063	0.7033	0.4560	0.063*	
O2B	0.45974 (15)	0.56659 (12)	0.12824 (10)	0.0530 (3)	
O3B	0.59711 (17)	0.75707 (12)	0.11953 (9)	0.0553 (4)	
C1B	0.51958 (18)	0.34408 (15)	0.25490 (12)	0.0342 (4)	
C2B	0.66394 (19)	0.21585 (15)	0.19986 (12)	0.0357 (4)	
C3B	0.6778 (2)	0.09090 (17)	0.16220 (14)	0.0482 (5)	
H3B	0.5944	0.0184	0.1500	0.058*	
C4B	0.8116 (3)	0.07581 (18)	0.14373 (16)	0.0560 (5)	
H4B	0.8196	−0.0072	0.1191	0.067*	
C5B	0.9382 (3)	0.1835 (2)	0.16113 (18)	0.0607 (6)	
H5B	1.0297	0.1717	0.1483	0.073*	
C6B	0.9281 (2)	0.30528 (18)	0.19672 (16)	0.0532 (5)	
H6B	1.0125	0.3764	0.2074	0.064*	
C7B	0.79075 (19)	0.32456 (15)	0.21766 (13)	0.0369 (4)	
C8B	0.77249 (18)	0.44774 (15)	0.25652 (12)	0.0364 (4)	
H8B	0.8541	0.5215	0.2686	0.044*	
C9B	0.63788 (17)	0.46076 (14)	0.27667 (11)	0.0316 (3)	
C10B	0.62325 (18)	0.59300 (14)	0.32479 (12)	0.0328 (4)	
H10B	0.5155	0.5844	0.3250	0.039*	
C11B	0.67326 (18)	0.68846 (14)	0.26558 (12)	0.0335 (4)	
C12B	0.7998 (2)	0.78554 (17)	0.29927 (15)	0.0470 (4)	
H12C	0.8246	0.8416	0.2595	0.056*	
H12D	0.8646	0.7982	0.3629	0.056*	
C13B	0.5651 (2)	0.66278 (15)	0.16500 (13)	0.0375 (4)	
C14B	0.4924 (3)	0.7379 (2)	0.02265 (16)	0.0765 (7)	
H14D	0.5241	0.8104	−0.0041	0.115*	
H14E	0.3909	0.7304	0.0321	0.115*	
H14F	0.4924	0.6599	−0.0246	0.115*	

### Atomic displacement parameters (Å^2^)

**Table:**

	*U*^11^	*U*^22^	*U*^33^	*U*^12^	*U*^13^	*U*^23^
Cl1A	0.0291 (2)	0.0501 (3)	0.0697 (3)	0.00871 (19)	0.0111 (2)	−0.0073 (2)
O1A	0.0485 (7)	0.0334 (6)	0.0626 (8)	0.0161 (6)	0.0061 (6)	0.0011 (6)
O2A	0.0628 (9)	0.0374 (8)	0.0763 (10)	−0.0048 (6)	0.0039 (8)	0.0076 (7)
O3A	0.0484 (8)	0.0571 (9)	0.0545 (8)	−0.0067 (6)	−0.0094 (7)	0.0015 (7)
N1A	0.0342 (7)	0.0321 (7)	0.0354 (7)	0.0073 (6)	0.0065 (6)	0.0029 (6)
C1A	0.0290 (8)	0.0363 (9)	0.0329 (8)	0.0085 (7)	0.0059 (7)	0.0052 (7)
C2A	0.0332 (9)	0.0356 (9)	0.0300 (8)	0.0054 (7)	0.0042 (7)	0.0093 (7)
C3A	0.0472 (11)	0.0356 (9)	0.0436 (10)	0.0040 (8)	0.0095 (8)	0.0079 (8)
C4A	0.0425 (11)	0.0462 (11)	0.0518 (11)	−0.0086 (9)	0.0031 (9)	0.0129 (9)
C5A	0.0308 (9)	0.0623 (13)	0.0565 (12)	0.0009 (9)	0.0039 (8)	0.0225 (10)
C6A	0.0335 (9)	0.0544 (11)	0.0499 (11)	0.0135 (8)	0.0089 (8)	0.0166 (9)
C7A	0.0326 (8)	0.0394 (9)	0.0323 (8)	0.0097 (7)	0.0046 (7)	0.0122 (7)
C8A	0.0359 (9)	0.0357 (9)	0.0355 (9)	0.0129 (7)	0.0089 (7)	0.0075 (7)
C9A	0.0322 (8)	0.0316 (8)	0.0301 (8)	0.0085 (7)	0.0063 (6)	0.0057 (6)
C10A	0.0358 (9)	0.0288 (8)	0.0383 (9)	0.0085 (7)	0.0101 (7)	0.0042 (7)
C11A	0.0329 (9)	0.0328 (9)	0.0377 (9)	0.0039 (7)	0.0079 (7)	0.0024 (7)
C12A	0.0561 (12)	0.0461 (11)	0.0455 (11)	−0.0004 (9)	−0.0049 (9)	0.0104 (9)
C13A	0.0383 (10)	0.0398 (10)	0.0422 (10)	0.0030 (8)	0.0108 (8)	−0.0001 (8)
C14A	0.0527 (13)	0.0717 (15)	0.0651 (14)	−0.0098 (11)	−0.0082 (11)	−0.0158 (12)
Cl1B	0.0352 (2)	0.0458 (3)	0.0787 (4)	0.0054 (2)	0.0187 (2)	0.0156 (2)
N1B	0.0413 (8)	0.0290 (7)	0.0403 (8)	0.0030 (6)	0.0077 (6)	0.0087 (6)
O1B	0.0493 (7)	0.0388 (7)	0.0343 (6)	0.0170 (6)	0.0034 (5)	0.0026 (5)
O2B	0.0555 (8)	0.0369 (7)	0.0473 (7)	0.0006 (6)	−0.0073 (6)	0.0035 (6)
O3B	0.0787 (10)	0.0391 (7)	0.0404 (7)	0.0100 (7)	0.0008 (6)	0.0144 (6)
C1B	0.0328 (8)	0.0326 (9)	0.0348 (9)	0.0054 (7)	0.0058 (7)	0.0110 (7)
C2B	0.0450 (10)	0.0278 (8)	0.0326 (8)	0.0078 (7)	0.0078 (7)	0.0093 (7)
C3B	0.0625 (12)	0.0281 (9)	0.0502 (11)	0.0083 (8)	0.0157 (9)	0.0068 (8)
C4B	0.0755 (14)	0.0349 (10)	0.0615 (13)	0.0233 (10)	0.0231 (11)	0.0080 (9)
C5B	0.0591 (13)	0.0498 (12)	0.0796 (15)	0.0261 (10)	0.0275 (11)	0.0107 (11)
C6B	0.0438 (11)	0.0393 (10)	0.0747 (14)	0.0113 (8)	0.0193 (10)	0.0088 (9)
C7B	0.0401 (9)	0.0314 (8)	0.0385 (9)	0.0105 (7)	0.0092 (7)	0.0085 (7)
C8B	0.0337 (9)	0.0264 (8)	0.0430 (9)	0.0032 (7)	0.0061 (7)	0.0065 (7)
C9B	0.0330 (8)	0.0287 (8)	0.0307 (8)	0.0065 (7)	0.0044 (7)	0.0090 (6)
C10B	0.0308 (8)	0.0291 (8)	0.0348 (9)	0.0074 (6)	0.0047 (7)	0.0051 (7)
C11B	0.0350 (9)	0.0252 (8)	0.0367 (9)	0.0089 (7)	0.0063 (7)	0.0033 (7)
C12B	0.0444 (10)	0.0370 (10)	0.0510 (11)	0.0017 (8)	0.0039 (8)	0.0124 (8)
C13B	0.0450 (10)	0.0274 (8)	0.0376 (9)	0.0121 (8)	0.0072 (8)	0.0047 (7)
C14B	0.115 (2)	0.0619 (14)	0.0432 (12)	0.0268 (14)	−0.0072 (12)	0.0172 (10)

### Geometric parameters (Å, º)

**Table:**

Cl1A—C1A	1.7396 (16)	Cl1B—C1B	1.7459 (17)
O1A—C10A	1.4228 (19)	N1B—C1B	1.295 (2)
O1A—H1A	0.8200	N1B—C2B	1.366 (2)
O2A—C13A	1.202 (2)	O1B—C10B	1.4130 (18)
O3A—C13A	1.322 (2)	O1B—H1B	0.8200
O3A—C14A	1.447 (2)	O2B—C13B	1.195 (2)
N1A—C1A	1.295 (2)	O3B—C13B	1.331 (2)
N1A—C2A	1.369 (2)	O3B—C14B	1.441 (2)
C1A—C9A	1.415 (2)	C1B—C9B	1.410 (2)
C2A—C3A	1.404 (2)	C2B—C7B	1.406 (2)
C2A—C7A	1.410 (2)	C2B—C3B	1.408 (2)
C3A—C4A	1.358 (3)	C3B—C4B	1.351 (3)
C3A—H3A	0.9300	C3B—H3B	0.9300
C4A—C5A	1.397 (3)	C4B—C5B	1.399 (3)
C4A—H4A	0.9300	C4B—H4B	0.9300
C5A—C6A	1.353 (3)	C5B—C6B	1.358 (3)
C5A—H5A	0.9300	C5B—H5B	0.9300
C6A—C7A	1.410 (2)	C6B—C7B	1.409 (2)
C6A—H6A	0.9300	C6B—H6B	0.9300
C7A—C8A	1.406 (2)	C7B—C8B	1.410 (2)
C8A—C9A	1.357 (2)	C8B—C9B	1.359 (2)
C8A—H8A	0.9300	C8B—H8B	0.9300
C9A—C10A	1.505 (2)	C9B—C10B	1.514 (2)
C10A—C11A	1.511 (2)	C10B—C11B	1.509 (2)
C10A—H10A	0.9800	C10B—H10B	0.9800
C11A—C12A	1.315 (3)	C11B—C12B	1.313 (2)
C11A—C13A	1.485 (2)	C11B—C13B	1.481 (2)
C12A—H12A	0.9300	C12B—H12C	0.9300
C12A—H12B	0.9300	C12B—H12D	0.9300
C14A—H14A	0.9600	C14B—H14D	0.9600
C14A—H14B	0.9600	C14B—H14E	0.9600
C14A—H14C	0.9600	C14B—H14F	0.9600
			
C10A—O1A—H1A	109.5	C1B—N1B—C2B	117.42 (14)
C13A—O3A—C14A	116.51 (17)	C10B—O1B—H1B	109.5
C1A—N1A—C2A	117.75 (14)	C13B—O3B—C14B	115.36 (15)
N1A—C1A—C9A	126.31 (15)	N1B—C1B—C9B	126.51 (15)
N1A—C1A—Cl1A	115.01 (12)	N1B—C1B—Cl1B	114.43 (12)
C9A—C1A—Cl1A	118.68 (12)	C9B—C1B—Cl1B	119.06 (12)
N1A—C2A—C3A	119.28 (15)	N1B—C2B—C7B	121.77 (14)
N1A—C2A—C7A	121.07 (14)	N1B—C2B—C3B	119.00 (15)
C3A—C2A—C7A	119.64 (15)	C7B—C2B—C3B	119.23 (16)
C4A—C3A—C2A	119.69 (18)	C4B—C3B—C2B	120.34 (18)
C4A—C3A—H3A	120.2	C4B—C3B—H3B	119.8
C2A—C3A—H3A	120.2	C2B—C3B—H3B	119.8
C3A—C4A—C5A	121.23 (17)	C3B—C4B—C5B	120.76 (17)
C3A—C4A—H4A	119.4	C3B—C4B—H4B	119.6
C5A—C4A—H4A	119.4	C5B—C4B—H4B	119.6
C6A—C5A—C4A	120.03 (17)	C6B—C5B—C4B	120.30 (19)
C6A—C5A—H5A	120.0	C6B—C5B—H5B	119.9
C4A—C5A—H5A	120.0	C4B—C5B—H5B	119.9
C5A—C6A—C7A	120.83 (18)	C5B—C6B—C7B	120.46 (18)
C5A—C6A—H6A	119.6	C5B—C6B—H6B	119.8
C7A—C6A—H6A	119.6	C7B—C6B—H6B	119.8
C8A—C7A—C6A	123.60 (16)	C2B—C7B—C8B	117.38 (15)
C8A—C7A—C2A	117.83 (15)	C2B—C7B—C6B	118.91 (15)
C6A—C7A—C2A	118.54 (15)	C8B—C7B—C6B	123.71 (16)
C9A—C8A—C7A	121.35 (15)	C9B—C8B—C7B	121.30 (15)
C9A—C8A—H8A	119.3	C9B—C8B—H8B	119.3
C7A—C8A—H8A	119.3	C7B—C8B—H8B	119.3
C8A—C9A—C1A	115.59 (14)	C8B—C9B—C1B	115.61 (14)
C8A—C9A—C10A	122.69 (14)	C8B—C9B—C10B	120.66 (14)
C1A—C9A—C10A	121.72 (14)	C1B—C9B—C10B	123.64 (14)
O1A—C10A—C9A	107.26 (13)	O1B—C10B—C11B	112.74 (13)
O1A—C10A—C11A	109.72 (13)	O1B—C10B—C9B	106.54 (12)
C9A—C10A—C11A	113.64 (13)	C11B—C10B—C9B	111.38 (13)
O1A—C10A—H10A	108.7	O1B—C10B—H10B	108.7
C9A—C10A—H10A	108.7	C11B—C10B—H10B	108.7
C11A—C10A—H10A	108.7	C9B—C10B—H10B	108.7
C12A—C11A—C13A	121.29 (16)	C12B—C11B—C13B	122.74 (16)
C12A—C11A—C10A	125.54 (15)	C12B—C11B—C10B	123.77 (15)
C13A—C11A—C10A	113.06 (14)	C13B—C11B—C10B	113.50 (13)
C11A—C12A—H12A	120.0	C11B—C12B—H12C	120.0
C11A—C12A—H12B	120.0	C11B—C12B—H12D	120.0
H12A—C12A—H12B	120.0	H12C—C12B—H12D	120.0
O2A—C13A—O3A	123.47 (17)	O2B—C13B—O3B	123.36 (15)
O2A—C13A—C11A	122.95 (17)	O2B—C13B—C11B	123.22 (15)
O3A—C13A—C11A	113.58 (16)	O3B—C13B—C11B	113.42 (14)
O3A—C14A—H14A	109.5	O3B—C14B—H14D	109.5
O3A—C14A—H14B	109.5	O3B—C14B—H14E	109.5
H14A—C14A—H14B	109.5	H14D—C14B—H14E	109.5
O3A—C14A—H14C	109.5	O3B—C14B—H14F	109.5
H14A—C14A—H14C	109.5	H14D—C14B—H14F	109.5
H14B—C14A—H14C	109.5	H14E—C14B—H14F	109.5
			
C2A—N1A—C1A—C9A	0.7 (2)	C2B—N1B—C1B—C9B	0.6 (2)
C2A—N1A—C1A—Cl1A	−179.69 (11)	C2B—N1B—C1B—Cl1B	−179.34 (11)
C1A—N1A—C2A—C3A	175.82 (15)	C1B—N1B—C2B—C7B	−0.9 (2)
C1A—N1A—C2A—C7A	−3.1 (2)	C1B—N1B—C2B—C3B	178.77 (15)
N1A—C2A—C3A—C4A	179.57 (16)	N1B—C2B—C3B—C4B	−179.50 (17)
C7A—C2A—C3A—C4A	−1.5 (2)	C7B—C2B—C3B—C4B	0.2 (3)
C2A—C3A—C4A—C5A	0.0 (3)	C2B—C3B—C4B—C5B	−0.3 (3)
C3A—C4A—C5A—C6A	1.2 (3)	C3B—C4B—C5B—C6B	−0.2 (3)
C4A—C5A—C6A—C7A	−0.7 (3)	C4B—C5B—C6B—C7B	0.7 (3)
C5A—C6A—C7A—C8A	177.30 (17)	N1B—C2B—C7B—C8B	0.4 (2)
C5A—C6A—C7A—C2A	−0.8 (3)	C3B—C2B—C7B—C8B	−179.26 (15)
N1A—C2A—C7A—C8A	2.6 (2)	N1B—C2B—C7B—C6B	−179.98 (16)
C3A—C2A—C7A—C8A	−176.31 (15)	C3B—C2B—C7B—C6B	0.3 (2)
N1A—C2A—C7A—C6A	−179.18 (15)	C5B—C6B—C7B—C2B	−0.8 (3)
C3A—C2A—C7A—C6A	1.9 (2)	C5B—C6B—C7B—C8B	178.76 (19)
C6A—C7A—C8A—C9A	−177.76 (16)	C2B—C7B—C8B—C9B	0.5 (2)
C2A—C7A—C8A—C9A	0.4 (2)	C6B—C7B—C8B—C9B	−179.11 (17)
C7A—C8A—C9A—C1A	−2.5 (2)	C7B—C8B—C9B—C1B	−0.8 (2)
C7A—C8A—C9A—C10A	178.19 (15)	C7B—C8B—C9B—C10B	176.02 (14)
N1A—C1A—C9A—C8A	2.1 (2)	N1B—C1B—C9B—C8B	0.3 (2)
Cl1A—C1A—C9A—C8A	−177.49 (12)	Cl1B—C1B—C9B—C8B	−179.82 (12)
N1A—C1A—C9A—C10A	−178.61 (16)	N1B—C1B—C9B—C10B	−176.42 (15)
Cl1A—C1A—C9A—C10A	1.8 (2)	Cl1B—C1B—C9B—C10B	3.5 (2)
C8A—C9A—C10A—O1A	14.0 (2)	C8B—C9B—C10B—O1B	−70.40 (18)
C1A—C9A—C10A—O1A	−165.25 (14)	C1B—C9B—C10B—O1B	106.14 (16)
C8A—C9A—C10A—C11A	−107.46 (17)	C8B—C9B—C10B—C11B	52.92 (19)
C1A—C9A—C10A—C11A	73.3 (2)	C1B—C9B—C10B—C11B	−130.54 (15)
O1A—C10A—C11A—C12A	−109.7 (2)	O1B—C10B—C11B—C12B	10.6 (2)
C9A—C10A—C11A—C12A	10.4 (2)	C9B—C10B—C11B—C12B	−109.15 (18)
O1A—C10A—C11A—C13A	66.52 (18)	O1B—C10B—C11B—C13B	−169.19 (12)
C9A—C10A—C11A—C13A	−173.42 (13)	C9B—C10B—C11B—C13B	71.10 (17)
C14A—O3A—C13A—O2A	−2.5 (3)	C14B—O3B—C13B—O2B	1.8 (3)
C14A—O3A—C13A—C11A	177.18 (17)	C14B—O3B—C13B—C11B	−178.39 (16)
C12A—C11A—C13A—O2A	164.7 (2)	C12B—C11B—C13B—O2B	170.78 (18)
C10A—C11A—C13A—O2A	−11.7 (2)	C10B—C11B—C13B—O2B	−9.5 (2)
C12A—C11A—C13A—O3A	−15.0 (3)	C12B—C11B—C13B—O3B	−9.0 (2)
C10A—C11A—C13A—O3A	168.60 (15)	C10B—C11B—C13B—O3B	170.72 (14)

### Hydrogen-bond geometry (Å, º)

**Table:**

*D*—H···*A*	*D*—H	H···*A*	*D*···*A*	*D*—H···*A*
O1*A*—H1*A*···O2*A*	0.82	2.24	2.8372 (19)	130
O1*B*—H1*B*···Cl1*A*^i^	0.82	2.79	3.5040 (12)	147
O1*B*—H1*B*···N1*A*^i^	0.82	2.16	2.8609 (17)	144
C5*A*—H5*A*···O1*B*^ii^	0.93	2.56	3.451 (2)	162

Symmetry codes: (i) −*x*+1, −*y*+1, −*z*+1; (ii) −*x*+2, −*y*+1, −*z*+1.
